# An Address on Medical Experts and Expert Testimony1Delivered by invitation before the Central New York Medical Association at its annual meeting held at Rochester, October 20, 1896.

**Published:** 1897-01

**Authors:** Frederick Sefton

**Affiliations:** Auburn, N. Y.


### BUFFALO MEDICAL JOURNAL.

VOL. XXXVI. JANUARY, 1897. No. 6.

### Original Communications.

### AN ADDRESS ON MEDICAL EXPERTS AND EXPERT TESTIMONY.[1]

BY FREDERICK SEFTON, M. D., Auburn, N. Y.

MAN'S mental activities for the last fifty years have been more absorbed in the pursuit of scientific knowledge than during the same period of any epoch touched by history. The world has been ripe for it, and the developments—one is tempted to say the creations—the result of this absorption have been phenomenal in their character and magnitude, and in their effects on the physical well-being of the individual as a unit, and on the complexion of society as a whole.

By means of them society practically has been reconstructed on new lines to its uttermost ramifications, and has become as distinct from the old order of things as was that order from aboriginal life.

To this onward rush and swirl of change, to this increase of complexity of life and living, our own profession, representative no longer of an art but a science, has in no small measure contributed. And thanks, if thanks be due, to universal education and the printing press, not alone the learned in other walks of life than ours, every one, the professor in his chair and the laborer in the ditch, has been awakened and claims some knowledge of the methods pursued, as well as of the results achieved.

As a corollary of this wonderful upgrowth of science, but perhaps chiefly because of the universal increase of knowledge of this upgrowth possessed by the masses, there has been created a new and vigorous bough on the already many-twigged tree of medical science. Rather a by-path, known to but few and by them seldom trod, has been transformed into a broad highway along which

1. Delivered by invitation before the Central New York Medical Association at its annual meeting held at Rochester, October 20, 1896.

many travel, some to fame, some, with regret it is admitted, to infame, and all to the temporary betterment of their financial standing. I refer to medical experts and expert testimony.

With the rise in importance of this branch of our practice, with the increase in frequency of its employment as a means of avoiding juries, or of influencing the verdicts of juries and the decisions of judges whenever avoidance has been deemed inexpedient or become impossible, it has been demonstrated to a conviction to all thinking professional men, legal as well as medical, that the accustomed methods employed in the production of lay testimony, or testimony as to facts, is unsuited to the proper development and presentation of this special class of evidence and that a radical change is necessary.

Many lawyers and some physicians believe if a change is not soon forthcoming, the employment of the latter as experts in courts of law will be relegated into "innocuous desuetude" by reason of the disgust of two of the parties interested, and to the permanent moral and financial injury of the third.

After some time spent in interviews and in correspondence with physicians in different localities, I have found few medical men of experience and standing who have not on more than one occasion been irritated, often have been chagrined and at times humiliated by their experiences in this line of work. Complaining justly that they never are permitted to state their findings to a jury in a logical, comprehensive manner, in a manner that would enable them fully and intelligently to embody all the essential medical facts in the case to the satisfaction of mind of the average juryman, but are bound down to "yes" and "no" replies to categorical questions, or forced in replying to utter trite commonplaces and well-worn stereotyped definitions, excellent sounding things, and well enough standing alone, yet completely failing to convey their real opinions, in fact any opinions, on the case immediately at issue. Or worse : forcing them by limiting the scope of the answers, to appear to give an opinion opposed to what they know to be the truth, or one which, while in no wise affecting their conclusions from a medical standpoint, to a lay jury seeming to be a retraction, even a contradiction, of testimony previously adduced by them. Or when an explanatory sentence would effectually clear a cloudy point they are forbidden to make it, at least at the time ; and if further along in the case so much of it as opposing counsel have agreed will not contravene the laws of evidence is haled in, it comes haltingly, from a physician's standpoint emasculated of all power to make fecund with understanding the mind of the normal juryman.

The existence of these conditions should make every one of us anxious for reform, and a reform that shall place the physician, as a witness in court, in the dignified position merited by the profession he has the honor to represent before a court of justice; that shall so restrict counsel in their dealings with experts as to make it impossible to frame an examination destined to parade a physician before court, jury and spectators as an interested, biased witness; one prone to exaggerate, eager to suppress facts against and to magnify out of all due proportions facts favoring the interests of the side in whose employ he chances to be, and, necessity arising, willingly, even cheerfully, perjuring himself for the sake of the remuneration promised.

This is not a pretty picture, but a true one. One held up to public view at nearly every trial at which a medical expert figures as a witness. One over the contemplation of which court and all honorable counsel lament, pettifoggers chuckle, juries grow weary and apathetic and every physician worthy the name indignant and ashamed.

A system that makes possible the exhibition of such a picture, leads to the degradation of medical science in the interest of the legal profession. Less than a fortnight ago, in my own city, the Hon. Justice Werner, presiding in the case of Sunderlin against Hollister and Noble, was led to say, after the introduction of the testimony of the attending physician and the calling to the witness box of another physician to testify as an expert: "You get three or four medical witnesses on one side and three or four on the other, and they serve only to confuse the case. The physician who attended her, the litigant, said more than will all the rest."

It is not long ago that a judge at a trial in Washington openly charged that experts could be hired for $100 or $150 a day to swear to anything required. Another judge is quoted as saying that no expert could refrain from becoming a partisan. Which means, if it means anything, that medical men are daily violating their oaths and prostituting their knowledge at the behest of lawyers.

Abroad it would appear to be little better. A recent number of *London Nature* says:

The fact that scientific men, some of them even of high standing, can be procured to sustain the most contradictory views is, we have no hesitation in saying, hurtful to the interests of science, and tends to degrade science in the opinion of the public.

The London *Times* thinks :

An expert reporting as a delegate of the Court would sometimes express himself very differently from one paid for his testimony.

Lawyers assert, and the assertion is plausible, that expert testimony procured at much pains and at great cost to the state and to the individual litigant, fails too often to subserve what should be its purpose—to so illumine disputed medical points with the light of true science that they shall shine forth clear and distinct to the mind's eye of the simplest—and rather throws an impenetrable veil of professional nomenclature over them, confusing judge, and so effectually befogging jury that in despair of ever comprehending, it casts out of the case all medical testimony and brings in a verdict based on the balance of the submitted evidence.

Yet that the bar complains might be thought slightly inconsistent. Certainly it becomes a trifle difficult to understand, when pausing to consider it is the application of rules and methods of which practically they themselves are the authors, that has begot the very evils they with us seemingly are anxious to eradicate.

Gentlemen—We have the diagnosis fairly complete ; we have ventured to suggest a prognosis if the disease remain unchecked ; what shall be the treatment ?

*First*—The medical expert himself: what shall be his qualifications ? To avoid the charge of presumption in discussing this point, it seemed advisable to quote other authority than mine, advancing my personal views only by inference to be drawn from the character of the matter quoted.

In France, in England or in Germany the courts would not recognise as an expert alienist a physician who had not at least several years of experience in special practice and study among the insane. Here, the ease with which anybody who wishes to call himself an expert may impose upon courts and juries is astonishing. His right to be considered so is never questioned. This is the reason that experts are so readily found who will swear that a sane man is a lunatic, that a notorious lunatic is in sound mind and all his delusions facts, that a man conducting his affairs perfectly has paresis and epilepsy and that another who clearly has paresis is capable of attending to his business as usual.

The *Medical News* of Philadelphia defines as its idea of an expert a man who :

Is above the level of the zeal of the partisan, the rage for publicity and the greed for lucre. . . . . So we have two kinds of experts, the first and greater of these stands for study, for observation, for candor, for science, for justice and for professional tradition. The second and lesser of these stands for rumor, for guesswork, for sensationalism, for newspaper notoriety and, above all, for self.

A daily New York newspaper says editorially :

The evils of pseudo-expertism must be indeed far-reaching when the organs of the medical profession are called upon to rebuke it. There is a prevailing lack of belief in experts nowadays among laymen, judges and juries. It is evident there must be a radical reform of some kind before the testimony of expert alienists in any given case can be accepted as always candid, honest and scientific. So long as any physician can pose as an expert if he thinks it profitable, so long as lawyers from either side may summon experts prejudiced in their favor by promise of payment for testlmony in behalf of one side, just so long will expert opinions be received with incredulity, and perhaps with contempt and ridicule by the public and by the juries. The medical profession owes it to itself to see that the law regarding the manner of selecting experts is changed, and to repudiate all sham pseudo-experts.

A law might be framed compelling every physician presenting himself to the court as an expert, to prove he possesses special qualifications for the work. First, that he is a regularly graduated physician and in good standing. Second, that he has had at least five years' practical experience along the particular lines presented in the case about to be the subject of judicial investigation. We must admit, such a law, though a vast stride toward the goal sought, alone would fail to bring about the desired end, though ever so thoroughly and conscientiously enforced. The sore needs more drastic treatment. We needs must go farther and remove the chief cause; or, that being impracticable, we must deprive him of his power for evil. And as a hair of the dog is said to cure the bite, I have gone to him for the remedy, and in doing so have demonstrated the truth of the adage once more. For, in my opinion, were the remedies he proposes employed they would right this grievous wrong.

The principal part of what follows is based on interviews with judges of the courts and members of the bar :

*First*—A justice of the Supreme Court of the state and a number of lawyers have informed me it was the determination of many members of the bar to agree to employ but two physicians, one for each side of a case, to limit expenses and minimise chances for confusion. Such a measure is not remedial. It fails to take cognisance of, to our profession, the worst features. It fails to secure to the physician adequate opportunities for a proper physical examination of the patient. It still permits counsel to throttle the physician on the witness stand as often as the exigencies of his side of the case may seem to demand.

*Second*—Lawyers propose to do away with expert testimony entirely and to rely on testimony as to facts, as was formerly done. This proposition, I am told, is especially enticing to those indolent and microcephalic disciples of Blackstone to whom the compilation of medical expert evidence is as the *pons asinorum* of their professional duties. In this advanced age such a reform is impossible, if advisable. The medical expert in one form or another has come to stay.

*Third*—It has been suggested that a law be enacted "requiring the appointment by the judge, of a commission of one to three experts to examine and report to him upon any medical question arising in the case." Such a change might be an improvement on existing methods, yet several objections at once suggest themselves to the mind. There would be danger that such an act would give rise to a set of court favorites composed of political doctors, so-called. If not, the difficulty of making a proper selection would be great; "for by what means would the judge be enabled to distinguish the true expert from the false?"

*Fourth*—A law might be passed compelling opposing counsel to meet and unite in choosing a single expert, as now they choose a referee. Such a law would have many advantages. Still, from the nature of the evidence demanded of an expert, called upon as he is not only for a statement of facts, but for conclusions based on those facts, he would be almost invariably at variance with one of the counsel, and be placed in as awkward and disagreeable a position as under the present methods. Besides, it might happen in important cases, it would be difficult, perhaps impossible, to secure a competent physician who would be willing to assume the responsibilities of the case alone. And there is wisdom, as well as safety to the doctor, in numbers.

*Fifth*—Each counsel might be compelled to present the names of three competent practitioners to the court, whose duty it should be to select one name from each side, and to add a third of his own choosing, the third not to be one of the original six; the three selected to constitute a commission, to whom every possible facility should be given for making a thorough scientific examination of the patient, even permitting them to summon and examine witnesses if necessary ; counsel on both sides to have free access to the commission for purposes of consultation ; the commission to make a full report in writing to the court, and at the trial to testify in open court ; the examination and cross-examination by counsel to be limited in its scope to the specifications contained in the written report in the hands of the presiding judge.

I have been requested to say a word before closing anent the woman medical expert. She is all right. But, as to practical workings under a special enactment. A law of this state, as you all know, in effect says : If counsel demand a physical examination of a female patient, the subject of litigation, she is privileged to refuse to be examined by any but a physician of her own sex, and if she does refuse, and an examination is insisted upon, counsel are compelled to engage the services of a woman physician for the purpose.

The law was enacted with the sole purpose, as I understand it, of conserving the modesty of the litigant. Such an object is most commendable. The law is defective, however, and its shortcomings were so well exemplified in a recent case with which I chanced to be connected, I can do no better, perhaps, than briefly to refer to it. The case, one of a married woman, the mother of several grown-up children, was approaching trial for the second time. At the time of the first trial a physical examination necessitating the exposure of her whole person had been made by several medical men of her own choosing, and by a woman physician and two male physicians, chosen by counsel for the defense. Between the first and second trials sufficient time had elapsed to effect a change. Perhaps she could not

> "Bid the cheek be ready with a blush,
> Modest as morning when she coldly eyes
> The youthful Phœbus,"

But she had progressed ; heedless that Seneca had said : "When modesty is once extinguished, it knows not a return," and having permitted five medical men, chosen by her counsel, to make the second examination, she refused to unveil herself to the brutal scrutiny of defendants' medical men, and compelled the defense to rest satisfied with an examination made by a woman physician.

It is proposed to have this law so amended that when a woman has practically waived her right to be examined by women physicians by submitting herself to examination by men physicians, chosen by herself or counsel, she shall be compelled to grant equal privileges to men physicians chosen by opposing counsel.

